# A randomized, prospective trial to assess the safety and efficacy of hilotherapy in patients after orthognathic surgery

**DOI:** 10.1007/s10006-021-00948-w

**Published:** 2021-03-05

**Authors:** Lars Bonitz, Adrian El-Karmi, Johannes Linssen, Dietmar Abel, Stefan Hassfeld, Ákos Bicsák

**Affiliations:** 1 Department of Oral and Maxillofacial Surgery, Dortmund General Hospital, Muensterstrasse 240, D-44145 Dortmund, Germany; 2 Department of Health, University Witten/Herdecke, Alfred-Herrhausen-Strasse 45, 58453 Witten, Germany

**Keywords:** Orthognathic surgery, Hilotherapy, Cooling temperature, 3D optical scanner, Swelling, Pain

## Abstract

**Purpose:**

A post-operative cooling method in oral and maxillofacial surgery is the cooling with hilotherapy. The aim of this study was the post-operative comparison of cooling temperatures of 18°C and 22°C. The parameters of this trial were swelling and the post-operative pain levels.

**Methods:**

This study included 156 patients, divided into two groups among whom a mono-one, bignathic osteotomy or genioplasty was indicated. The post-operative assessment of swelling was performed using a 3D optical scanner. This examination was repeated on post-operative days 1, 2, 3, 7, 14, 30, and 90. The examination on day 90 served as a reference value in respect of swelling and pain.

**Results:**

Group 1 (18°C, 78 patients) showed an increase in post-operative swelling on the 1^st^ post-OP day of 52.06 ± 35.41ml. The maximum was reached on the 2^nd^ post-OP day with 75.82 ± 38.97ml. On the 30^th^ post-OP day, residual swelling measured 11.60 ± 12.62ml. Group 2 (22 °C, 78 patients) showed an increase in postoperative swelling on the 1^st^ post-OP day of 76.07 ± 63.15ml. The maximum was reached on the 2^nd^ post-OP day with 106.97 ± 69.63 ml. On the 30^th^ post-OP day, residual swelling measured 14.36 ± 32.26ml. The differences between the two groups and between different visits were statistically significant.

**Conclusion:**

The study results indicate less residual swelling in group 1 on the 30^th^ post-OP day, possible based on the lower cooling temperature. The post-operative pain exhibits a comparable level of pain intensity between the two groups. In overall terms, a subjectively more agreeable treatment was observed in group 1.

## Introduction

Local swelling, hyperemia, hyperthermia, pain, and altered function are well-known consequences of tissue injuries [1]. Reduction of all of these leads to the enhanced healing process, decreasing of pain, and improved post-injury or postoperative quality of life [2–7]. The swelling reaches its maximum after approximately 48 to 72 h after the operation [7, 8]. In this period, it is essential to provide adequate pain medication (non-steroidal antirheumatic drugs—NSAIDs [9], corticosteroids [10], or enzymatic drugs [11]) and is highly suggested to provide supportive local therapy, like manual lymph drainage [12], cooling with wet tissues or cryotherapy [2, 7, 13, 14].

Results have shown that nerve conduction speed drops with decreasing temperature [15, 16] that decreases the sensitivity of the nociception until a total blockade and thus analgesia at approximately 15°C are reached [17]. At temperatures below 10°C, the risk of tissue damage increases [18–20]. Between 20 and 23°C, this drop of the excitability is linear; below this temperature, the raise of this threshold is rapid [21]; and at 4°C, it reaches a complete blockade [16]. By reducing the impulse frequency generated in the axons by influencing membrane properties, it is possible to modify the pain sensation [22]. Cryotherapy reduces the release of inflammatory mediators and enzymatic activity in the local tissue; thus, it leads to a decrease of hematoma and edema formation, too [23]. In oral and maxillofacial surgery, cryotherapy has become a proven supportive therapy after the removal of third molars [3, 8, 10, 14, 23, 24], traumas [25, 26], or after orthognathic surgery [5, 7, 25].

The Hilotherm Clinic device (Hilotherm® GmbH, Argenbühl-Eisenharz, Germany) provides a dry, easy-to-use, continuous cold therapy in a controlled manner that allows in clinical setup even patient-controlled cooling effect. This is reached by anatomically shaped sleeves for many body regions, including the face, in which tempered water is circulated. The setup allows temperature ranges from +10 to +35°C [7]. Hilotherapy is the term used in the literature for cryotherapy by using Hilotherm® devices [7, 25, 27–29].

## Purpose

This study aims to examine and compare the safety and efficacy of hilotherapy at two different cooling temperatures at 18°C or 22°C in an investigator-initiated, randomized, prospective, monocentric cohort study. The endpoints for the study were patient assessed subjective pain level and postoperative swelling measured by volume changes of the facial tissue at different visits. The parameters of this trial are swelling and the post-operative development of pain levels. Examining the efficacy of two different cooling temperatures can allow a more effective setup of postoperative pain therapy with a reduction of safety issues that include possible frost injuries, allergies against medicines, or other side effects, like gastric pain or ulcer.

## Material and methods

This study was examined and approved by the Ethics Committee at the Medical Faculty of the Westfalian Wilhelms-University in Münster, Germany (№ 2013-239-f-S). Before conducting any study procedures, written informed consent was obtained from all study subjects.

As baseline values, data from day 90 assessment was used. This is possible, as there were no early relapses diagnosed but all changes in hard and soft tissue configurations are stable. Also 90 days post operatively swelling is expected to be completely resolved.

The study was conducted in a semi-blinded manner. Patients were not aware of the cooling temperature (device digital screen showing temperature data was covered with black tape, an unblinding was not detected; therefore, this had no impact on study data). The investigator performing the volume measurements was not aware of cooling temperatures.

### Patients

We conducted this study at the Department of Cranio-, Maxillofacial Surgery, Regional Plastic Surgery at Dortmund General Hospital. We involved 156 subjects who underwent orthognathic surgery. The patients were divided into two randomized groups receiving hilotherapy at preset temperatures of 18°C or 22°C. The randomization was performed by using an online randomizer (www.randomizer.org). The surgical procedure included monomaxillary or bimaxillary osteotomy with or without genioplasty performed by the same surgeon. The indication of surgery was standard indication and did not influence the study procedure. The maxillary osteotomy was performed at the Le-Fort-I level (Reuther, 2000), whereas the mandibular split was performed as per Obwegeser and Trauner. Demographic data and surgery length were documented.

### Hilotherapy

**Fig. 1:**
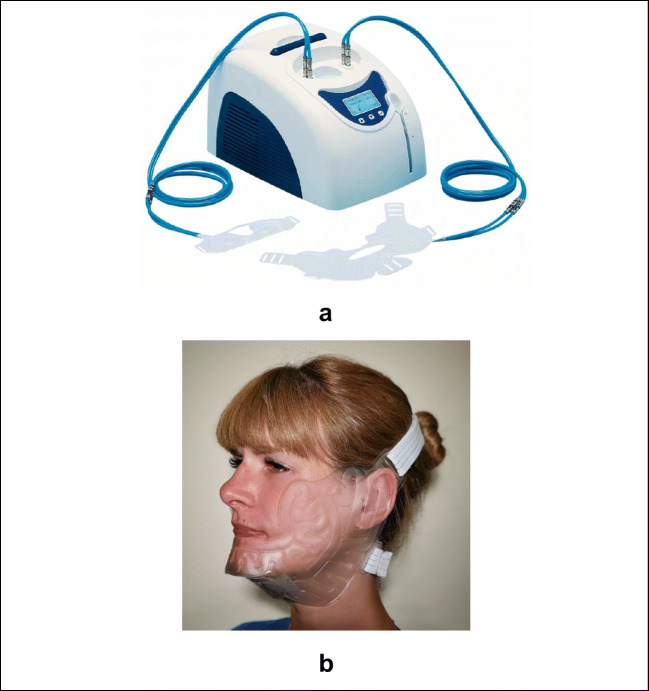
a Demonstration of the Hilotherm® Clinic device with an adjustable temperature range of +10 to +35°C. **b** Demonstration of the face sleeve applied to patients (Hilotherm® GmbH)

The hilotherapy was provided by the Hilotherm® Clinic device (Hilotherm® GmbH, Argenbühl Eisenharz, Germany). This enables a temperature-controlled water flow in the anatomically adapted and very lightweight facial sleeve (hypo-allergic, latex-free thermoplastic polyurethane sleeve). Through this continuous flow, a stable skin surface temperature was set at 18°C or 22°C (Fig. 1a, b).

### Inclusion, exclusion, and study parameters

Table 1 provides an overview of the inclusion and exclusion criteria. In accordance with the general perioperative procedure for orthognathic surgery at our department and as per study protocol, all patients received hydrocortisone 500mg i.v. once and antibiotics—3 g ampicillin-sulbactam i.v. (or if not compatible, clindamycin 600 mg i.v.)—was administered starting before the operations and continued every 8 h for 3 days postoperatively. Postoperatively ibuprofen 600mg three times a day p.o. was administered. The postoperative X-rays and lab tests were carried out as per clinic standards. Patients were discharged after 3 days of therapy. The antibiotic therapy was continued with amoxicillin/clavulanic acid 875/125mg (or 600mg clindamycin) p.o. three times a day up to 7 days. For pain therapy, ibuprofen 600 mg was advised up to 3× daily on demand and subject to compatibility. During the entire investigation period, swelling and pain levels were examined and documented based on the 6-point “visual analog scale” (VAS).

**Table 1:** Summary of study inclusion and exclusion criteria

Inclusion criteria	Exclusion criteria
Surgery for dysgnathia: bimaxillary osteotomy or monomaxillary osteotomy	Allergy, intolerance or contraindication of glucocorticoids or NSAID in the medical history
Signed study informed consent form	Non-compliance with the requested cooling therapy of at least 16 h/day
	Inability to participate
	Known contraindication of cooling therapy (e.g., Raynaud’s syndrome, cryoglobulines)

### Clinical trial procedure

Hilotherapy was introduced as soon as the patients were capable after the general anesthesia. The application of the different cooling temperatures was carried out in a single-blinded manner so that the patients were not informed about their settings. The daily application of hilotherapy lasted for at least 16 h. During the inpatient period, pain medication was administered as per plan.

**Fig. 2:**
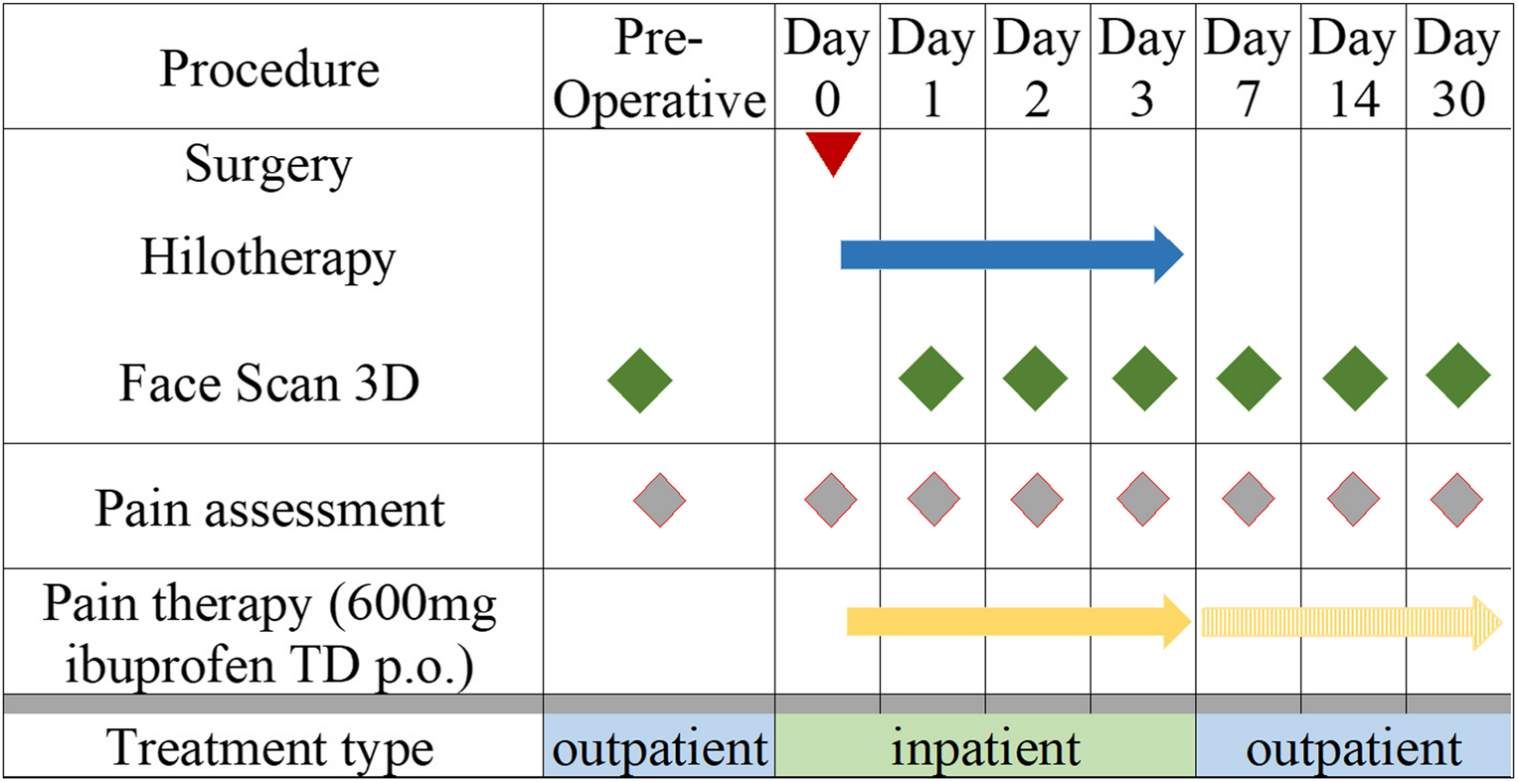
Summary of study procedures

The swelling assessment was conducted with a 3D optical scanner (FaceSCAN3D®). The pain levels were recorded by using a visual analog scale, VAS. This examination was repeated on post-operative days 1, 2, and 3 during the inpatient stage of this treatment. While being treated as in-patients, as well as a check on progress, a daily mouth and tooth cleaning procedure was followed. During the out-patient stage, the investigation times were post-operative days 7, 14, and 30 (Fig. 2).

### Assessment of facial swelling

The examination of post-operative swelling was performed with a 3D optical scanner (FaceSCAN3D®). The FaceSCAN3D® sensor was developed to record three-dimensional facial photographs and to perform volume measures based on these recordings. The sensors are based on the principle of strip projection. The distortion of these light strips is used to gain topographic information. This measurement process generates a 3D database and records the texture of the soft tissue surface (FaceSCAN3D®, 3D-Shape GmbH, Erlangen, Germany).

### Volumetric assessment

**Fig. 3:**
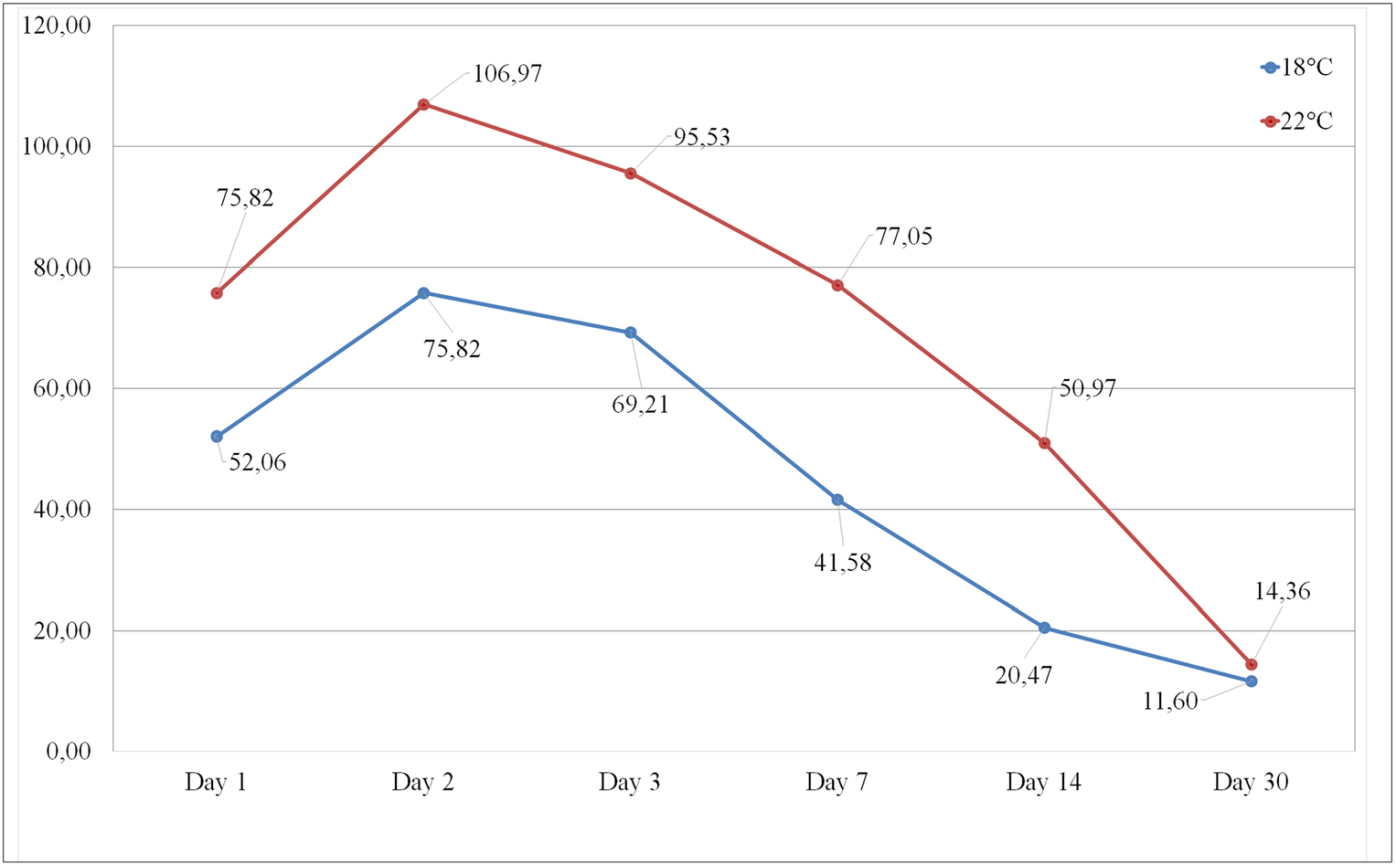
Comparison of the excess swelling in both patient groups (average; ml)

We used MIS (Mimics Innovation Suite) vers. 23 with 3matic vers. 16 to evaluate the three-dimensional datasets and to assess volumetric changes based on this information. As anatomical landmarks, we used the nasion and tragus on both sides, as they remain unchanged throughout all procedures (Fig. 3).

### Statistical analysis

The statistical analysis was conducted by an independent statistician using the statistical program R Development Core Team (2015). R: A language and environment for statistical computing. R Foundation for Statistical Computing, Vienna, Austria, Version 3.2.0. Using the non-parametric Wilcoxon-Mann-Whitney test (group comparison), the data were examined for signs of significant differences. A value of *p* < 0.05 was declared as statistically significant.

## Results

### Study patients and characteristics

This study included 156 patients who underwent orthognathic surgery at the Department of Cranio-, Maxillofacial Surgery, Regional Plastic Surgery at Dortmund General Hospital. The treatment response of both randomized patient groups at 18°C and 22°C was compared. Both randomized groups were treated in a standardized manner by means of hilotherapy. The basic characteristics of the subjects are presented in Table 2. All genioplasties were performed in connection with bimaxillary osteotomies.

**Table 2:** Summary of the patient group demographics

	Group 1—18°C	Group 2—22°C
Bimax:monomax:genioplasty	53:25:11	48:30:11
Patients (*n*)	78	78
Gender (W 69.5%/M 30.5%)	40/38	44/34
Age (years) ± SD	22.84 ± 6.42	21.98 ± 5.78
Duration of operation (min) ± SD	132.3 ± 51.1	115.1 ± 42.9
Duration of hospital stay (days)	4	4
Number of surgeries*	2/15/53/8	-/14/57/7

*The surgeries are listed as follows: LeFort I maxillary osteotomy/mandibular osteotomy/bimaxillary osteotomy/bimaxillary osteotomy and genioplasty

### Post-operative swelling

**Fig. 4:**
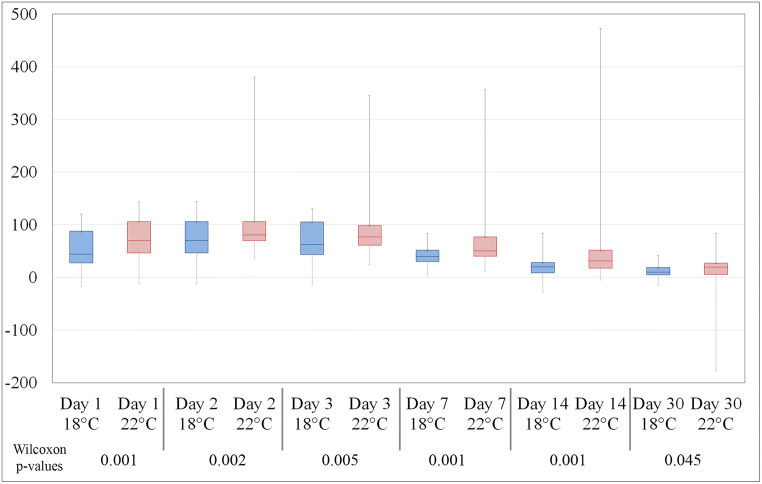
Comparison of swelling characteristics (boxplot diagrams show the swelling means, maximums, and minimums to different study milestones). The Wilcoxon *p*-values show a statistically significant difference up to 2 weeks postoperative. After a month time, the swelling is almost gone and there is no significant difference between the two treatment groups

The assessment of the swelling was performed on postoperative images. Day 90 volume data was taken as baseline. Both groups show similar postoperative swelling development (Figs. 3 and 4). On the 1^st^ post-operative day, group 1 (18°C) exhibits an increase of 52.06 ± 35.41 ml. The maximum level of swelling was reached on day 2 with 75.82 ± 38.97 ml. Then, the swelling reduced successively over time and there was no significant rest after 30 days postoperatively and measured 11.60 ± 12.62 ml (*p*=0.045).

**Fig. 5:**
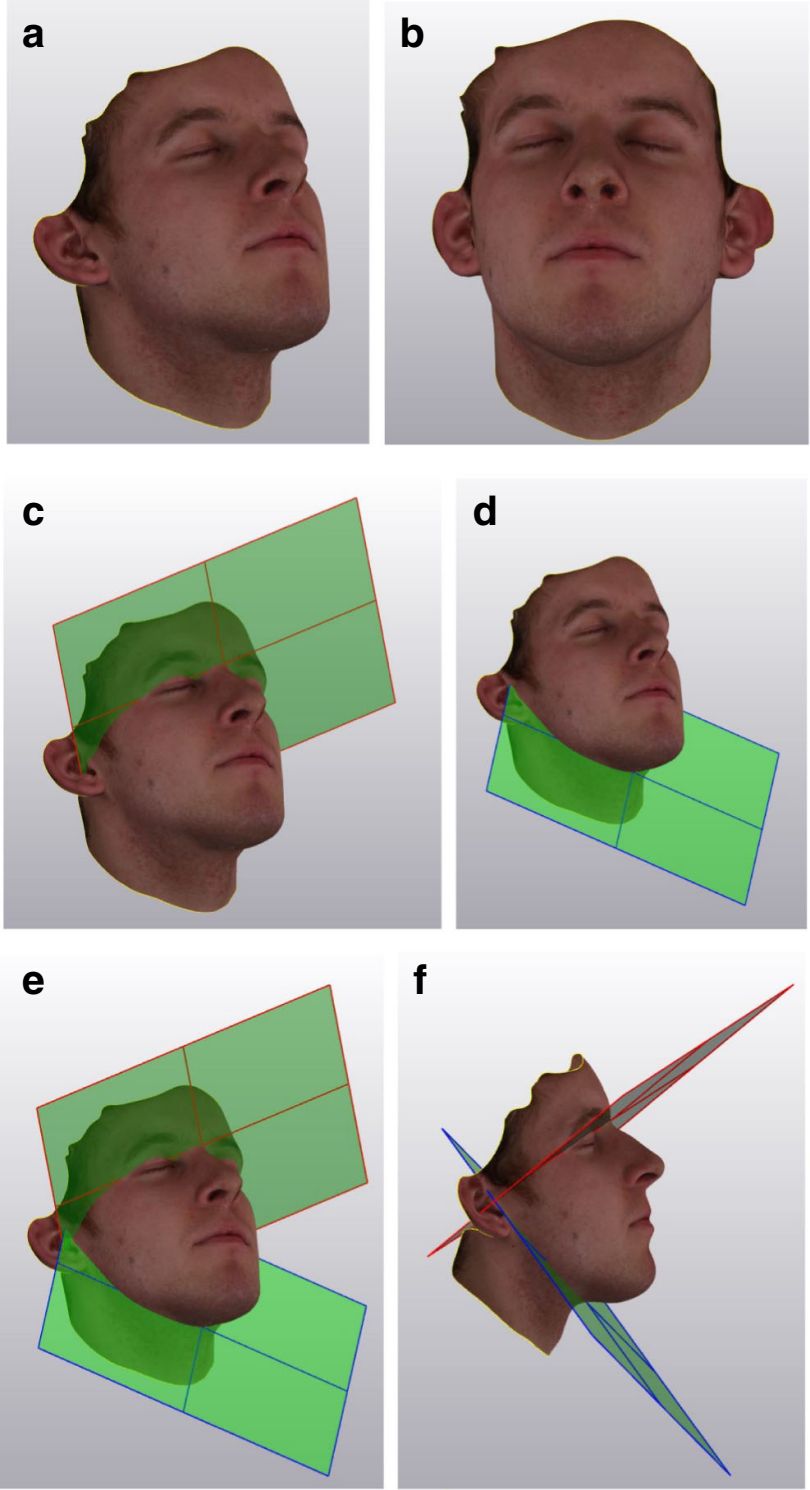
**a** 3D face scan, oblique right view. **b** 3D face scan, frontal view. **c** 3D face scan with cranial plane, oblique right view. **d** 3D face scan with caudal plane, oblique right view. **e** 3D face scan with segmented face areal by cranial and caudal planes, oblique right view. **f** 3D face scan with segmented face areal by cranial and caudal planes, right lateral view

Group 2 (22°C) showed during the whole study period much more swelling reaching 106.97 ± 69.63 ml on day 2 and ending with 14.36 ± 32.26 ml (*p*=0.045) after 30 days. The differences from day 1 to day 14 were statistically significant, but not on day 30 (Fig. 5).

### Intensity of pain

**Fig. 6:**
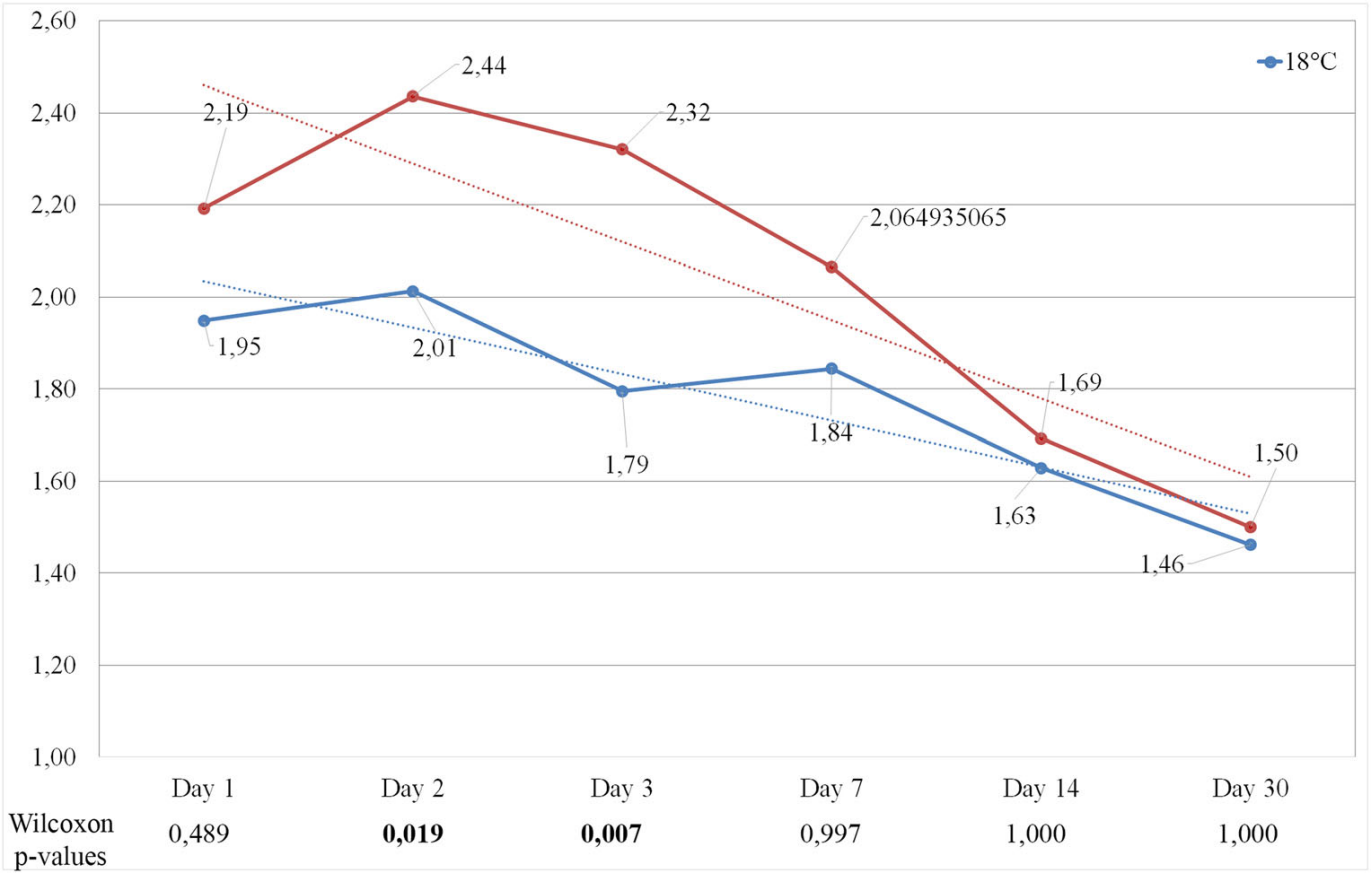
Presentation of the subjective pain perceptions on visual analogue scale (VAS) in both treatment groups. Wilcoxon *p*-values show a significant difference on day 2 and day 3 during the hilotherapy, but not before or after its application. Significant values are highlighted in bold; trend lines are presented in dotted lines

Post-operative pain was assessed using the 10-point visual analog scale (VAS) during the study period. Both groups showed the most pain on day 2; however, group 1 had much less pain than group 2 (2.01 vs. 2.44 VAS score respectively). After that, the trend is decreasing and there is no significant difference after finishing the hilotherapy. On the day of the surgery, group 1 showed remarkably less pain, but this difference was not significant (Fig. 6).

## Discussion

This study examined 156 patients, who received hilotherapy at 18°C and 22°C after the orthognathic surgery procedure. The most important study endpoints were postoperative swelling and pain. Our results have shown that hilotherapy contributes effectively to decrease both postoperative swelling and pain, thus reducing the need for painkillers helping to avoid their side effects. This result corresponds with the literature data [7, 30]. We found further that the hilotherapy at the above temperature levels has an excellent safety profile. We did not register any side effects of hilotherapy. Some authors reported neuropraxia that leads to paresthesia, e.g., paradox vasodilatation, bradycardia, elevated blood pressure, and increased cerebral blood flow through changes of the activation levels and balance between the sympathetic and parasympathetic system [31, 32].

One of the most important findings of this study was the onset and characteristics of the postoperative soft tissue swelling of the face. The patient group with a temperature of 18°C showed a slower onset, the lower peak of swelling, with the maximum level of swelling on day 2. In the group that used 22°C temperature, the peak was higher and the onset accelerated and reached a maximum on day 2 after surgery. The decrease of the postoperative edema showed a similar characteristic: at the lower temperature, the decrease was faster, and the residual swelling was less than in the other group. This result was statistically significant and corresponds with those of Rana et al. [5] and Modabber et al. [6] and El-Karmi et al. [7]. Hilotherapy effectively normalizes local blood flow that regulates fluid extravasation, inflammatory reaction, pain, and the activation of the lymphatic system [17]. Rana et al. confirmed the superiority of hilotherapy over the traditional cooling with wet tissues [5]. The literature states the superiority of hilotherapy over conventional cooling regarding patient satisfaction [7, 29]. There were no inconvenience issues reported by our patients.

The central unit of the device allows a step-by-step adaptation of the cooling temperature, which allows patient-controlled cryotherapy and can lead to further reduction of drug usage and complications. This requires another clinical trial to confirm this.

Limitation of this study is its unblinded nature. However, the volume assessment is digitally performed; thus, the unblinded investigator plays no role in data quality. Patients know in which group they are and VAS is a subjective measure; at this point, there is no more bias seen than in other studies.

## Conclusion

Hilotherapy is a proven method in postoperative pain reduction. It is convenient enough for daily clinical use, clean, dry, and easy to apply. Both 18°C and 22°C are safe temperatures for hilotherapy. Cooling at 18°C provides better pain and swelling control over time.
